# Deep vs shallow: GPS tags reveal a dichotomy in movement patterns of loggerhead turtles foraging in a coastal bay

**DOI:** 10.1186/s40462-024-00480-y

**Published:** 2024-05-30

**Authors:** Margaret M. Lamont, Daniel Slone, James P. Reid, Susan M. Butler, Joseph Alday

**Affiliations:** https://ror.org/05qtybq80 U.S. Geological Survey, Wetland and Aquatic Research Center, Gainesville, FL 32653 USA

**Keywords:** Gulf of Mexico, Telemetry, *Caretta caretta*, Home range, Diel

## Abstract

**Background:**

Individual variation in movement strategies of foraging loggerhead turtles have been documented on the scale of tens to hundreds of kilometers within single ocean basins. Use of different strategies among individuals may reflect variations in resources, predation pressure or competition. It is less common for individual turtles to use different foraging strategies on the scale of kilometers within a single coastal bay. We used GPS tags capable of back-filling fine-scale locations to document movement patterns of loggerhead turtles in a coastal bay in Northwest Florida, U.S.A.

**Methods:**

Iridium-linked GPS tags were deployed on loggerhead turtles at a neritic foraging site in Northwest Florida. After filtering telemetry data, point locations were transformed to movement lines and then merged with the original point file to define travel paths and assess travel speed. Home ranges were determined using kernel density function. Diurnal behavioral shifts were examined by examining turtle movements compared to solar time.

**Results:**

Of the 11 turtles tagged, three tracked turtles remained in deep (~ 6 m) water for almost the entire tracking period, while all other turtles undertook movements from deep water locations, located along edges and channels, to shallow (~ 1–2 m) shoals at regular intervals and primarily at night. Three individuals made short-term movements into the Gulf of Mexico when water temperatures dropped, and movement speeds in the Gulf were greater than those in the bay. Turtles exhibited a novel behavior we termed drifting.

**Conclusions:**

This study highlighted the value provided to fine-scale movement studies for species such as sea turtles that surface infrequently by the ability of these GPS tags to store and re-upload data. Future use of these tags at other loggerhead foraging sites, and concurrent with diving and foraging data, would provide a powerful tool to better understand fine-scale movement patterns of sea turtles.

**Supplementary Information:**

The online version contains supplementary material available at 10.1186/s40462-024-00480-y.

## Background

Animals move for a variety of reasons including migration, foraging, reproduction, dispersal and exploration. In the marine environment, animals live in heterogenous environments that often contain patchy resources [1, 2] therefore they must integrate physiological, ecological, and behavioral information to make daily movement decisions [3]. These movements can include long-distance migrations [4] and fine scale shifts in behavior due to changes in prey [5] or the presence of predators [6]. Much of the focus of animal movement studies has been on long-distance movement patterns [7, 8] and their connection to anthropogenic or climactic drivers [9]. For marine species in particular, quantifying fine-scale linkages between organisms and their resources has been more difficult. However, advances in biologging techniques can provide the detailed data needed to better understand those linkages [2, 10, 11].

Location data provided via both satellite-based Argos tags and acoustic telemetry generate spatial information for marine vertebrates, but each method has limitations. Error associated with locations from Argos tags makes it difficult to link fine-scale movements to environmental variables that drive those movements [12, 13] while acoustic telemetry data are limited to areas where receivers are present [14]. Acquisition of Global Positioning System (GPS) locations provides accurate spatial data that allows for high resolution examinations of movement patterns relative to an animal’s environment [1, 11]. Development of rapid acquisition of GPS data that is linked to the Argos system (e.g., Fastloc) has provided new insights into the fine-scale movements of marine organisms, including sea turtles, and can provide detailed data that spans months or even years [15]. Argos tags can store location data on the tag, allowing data to be transmitted for relatively long time periods (typically 10 days) after they are collected. However, the limited bandwidth of Argos (256 bits per uplink) means that limited amounts of data can be transmitted. Further, Argos tags typically have an external whip antenna which can be a point of weakness, shearing off so that data transmissions cease [16, 17]. Iridium tags provide a solution to some of these problems by containing a far greater bandwidth, allowing more data to be transmitted, and are capable of two-way messaging. If communication with the satellites is unavailable, the data are stored and then re-transmitted (i.e., backfilled) automatically at a later time when communication is restored, which is particularly useful for species such as sea turtles (Jim et al. 2022, Jang et al. 2024) that spend much of the time submerged or during times of high wave energy when transmitters have limited success at satellite communication [16, 18]. This feature is also available from Global Systems Mobile Communication (GSM) Cellular two-way tags however that technology relies on base stations to log data and as such would only be appropriate for terrestrial or coastal species (Matos et al. 2015). The downside to iridium tags is that they require significantly longer surface time compared to Argos tags to relay information (8.5 s vs. 0.3–0.9 s), although with two-way communication, receipt of the message is confirmed, while Argos tags repeat messages many times to improve the possibility of receipt. Additionally, Iridium tags have no external antenna which may reduce the frequency of satellite acquisitions but may also provide a benefit as breakage of external antennae is often a source of tag failure [15, 16]. As such, for some species, this amount of time at the surface may be insufficient for relaying location and other data via the Iridium network. Nevertheless, previous studies have shown that loggerhead turtles are an excellent candidate species for Iridium tracking [19–21]. Use of GPS telemetry has highlighted the patchiness of marine habitats [1, 22] and the complexity of factors that drive movement patterns of marine vertebrates [23]. Inclusion of Iridium-linked GPS tags, particularly with loggerhead turtles, could improve those studies.

Of particular interest is the understanding of movement patterns within an animal’s home range because these areas typically encompass the resources that are most critical to species survival [24–26]. In addition, information on home ranges is necessary for understanding ecological communities, determining location and size of marine protected areas and assessing the threat potential of an invasive organism [27–29]. For marine species, such as sea turtles, size and location of home ranges is frequently defined [4, 30, 31] however these characterizations have generally been conducted on the mesoscale level [4, 32, 33]. Fine-scale features, and the associated movements undertaken by the animal in response to them, are less well-known but have started to be revealed through Fastloc-GPS Argos tracking [15]. These movements are more difficult to measure and the fine-scale features, such as prey assemblages, are often less predictable [34], and these knowledge gaps can limit effectiveness of conservation actions [35].

Loggerhead sea turtles (*Caretta caretta*) establish both juvenile and adult foraging home ranges, which are often disparate but occasionally overlap [36]. These foraging sites are spatially and temporally patchy on multiple scales and this variability affects movements [1]. Variability in movements of juvenile and adult loggerheads have been documented, primarily in response to mesoscale features such as habitat use (e.g. oceanic versus neritic) and seasonal temperatures [13, 37]. Less is known about what drives fine-scale movements within juvenile loggerhead home ranges, particularly in areas also shared by adults. Here we use GPS tags to examine fine-scale movements of loggerhead turtles at a neritic foraging area. By closely examining daily movement patterns, we predicted turtles would move within their home ranges in response to environmental and habitat variables including water depth and tidal cycles.

## Methods

### Study site

St. Joseph Bay is located in northwest Florida in the northern Gulf of Mexico and encompasses approximately 260 km2. The greatest depths in the bay (max depth 13.3 m) are located in the middle and northern portions of the bay and consist primarily of sand and mud sediments, and the shallowest depths (~ 0.5 m) occur at the southern end [38]. Some of the most pristine seagrass beds in the state of Florida, dominated by *Thalassia testudinum*, fringe the majority of the bay, with extensive shoals covering the southern end [38]. Along the edges of the bay, these shallow (0.5–1.5 m) shoals are relatively narrow (~ 100–500 m) and drop off immediately into deeper (~ 6 m) waters. In the southern end, the expansive shoals are transected by a series of relatively deep (6–7 m) sandy-bottomed channels [39].

### Turtle captures and tagging

**Fig. 1:**
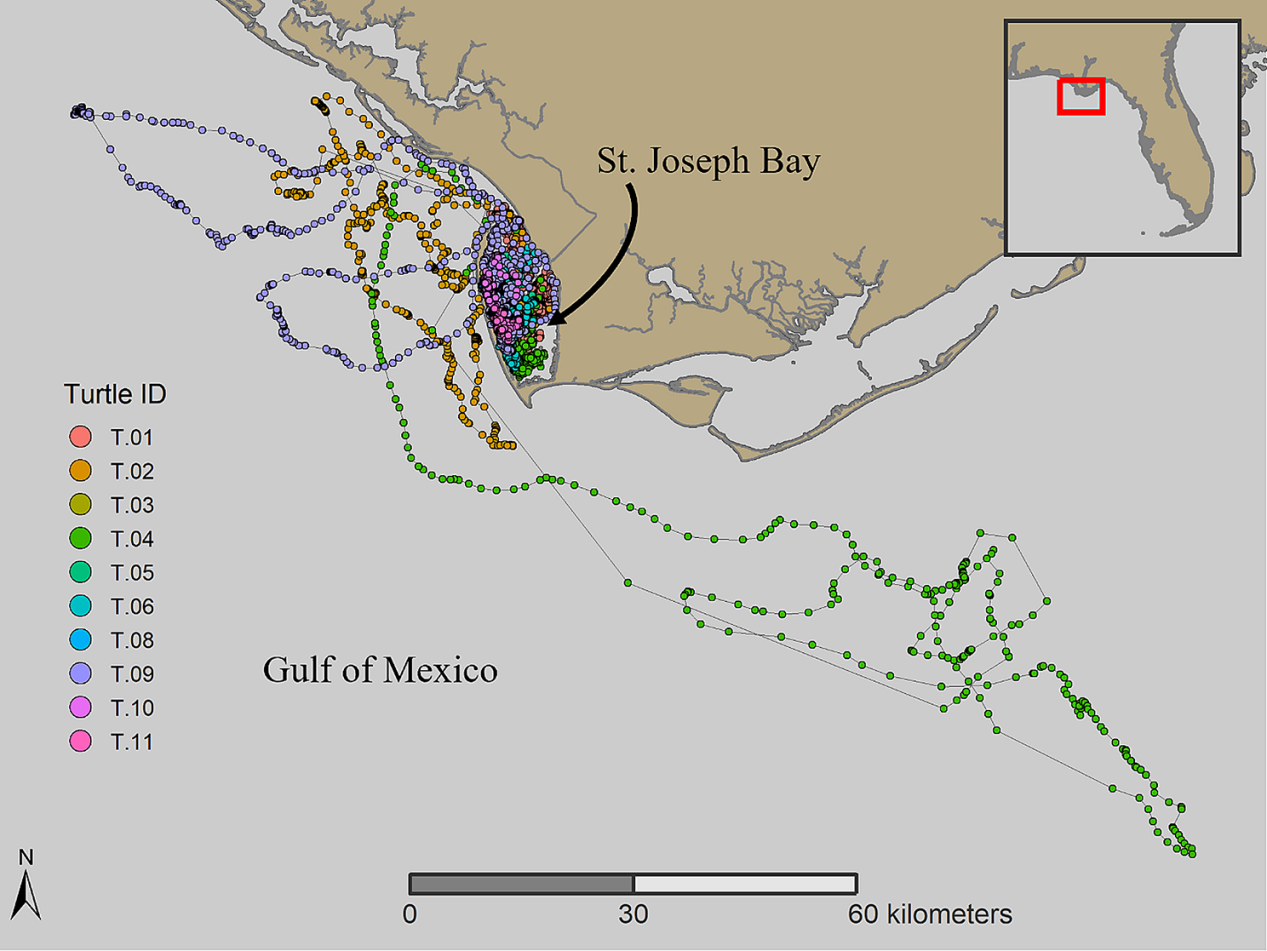
Map of St. Joseph Bay in northwest Florida showing the movements of 10 loggerhead sea turtles recorded hourly with GPS tags from September 2019 – September 2021

Loggerhead turtles (*n* = 11) were captured by hand from a 19-ft Boston Whaler in St. Joseph Bay, Florida between August 2019 and June 2021 (Fig. 1). After being observed from the vessel, personnel jumped from the boat, grabbed ahold of the rear carapace of the turtle and brought the individual to the surface before transferring the captured animal to the boat for tagging. All captured turtles were individually marked with a metal.

Inconel tag placed along the trailing edge of each front flipper and a passive integrated transponder (PIT) tag placed subcutaneously in the left shoulder. Curved carapace length (CCL) of turtles was measured using a cloth tape measure. Weight was determined by placing the turtle in a harness and hanging the harness from a hand-held Pesola spring scale. The entire work up and satellite tagging process took approximately 1.5 h and all tagged turtles were released at their original capture site.

To quantify fine-scale movements and activity patterns, the turtles were continuously tracked with Iridium-linked GPS tags (SeaTrkr 4370, Telonics Inc., Mesa AZ) that were affixed to the carapace using cool-setting epoxy (Superbond). Before deployment, tags were lightly sanded and covered with anti-fouling paint (Pettit Hydrocoat 1240 Blue, Modern Recreational Technologies, LLC, Hickory, NC, USA), as satellite tags deployed in the northern Gulf of Mexico fail primarily due to biofouling [16]. We streamlined tag attachment materials to minimize drag effects [40] on turtle’s swimming ability. These tags contained quick-fix pseudoranging (QFP) technology that were capable of obtaining a GPS position in an average of six seconds during our field testing on sea turtles, which is approximately six times faster than a conventional GPS location with very little reduction in accuracy. This technology provides a “middle ground” between full GPS and snapshot GPS receivers such as Fastloc which can provide a much faster location (a few 10’s of milliseconds for Fastloc-GPS tags) at the cost of greater positional error (Our QFP tags provided 50% location accuracy of 6 m and 90% accuracy of 15 m with 6 satellites compared to 18 and 70 m respectively for Fastloc; [41, 42], making it possible to get extremely high positional accuracy on species that surface long enough for this technology to work, such as loggerhead sea turtles. The tags were programmed to obtain exclusively QFP to extend battery life, with a frequency of one location per hour. Position data and hourly water temperature data were periodically uploaded to the Iridium (Iridium Communications Inc., McLean, Virginia) satellite network for archiving and accessed for analysis using Telonics Data Converter (v. 2.80, Telonics Inc., Mesa AZ).

### Data analysis

After downloading telemetry data with Telonics Data Converter, all further processing and analyses were produced with the statistical program R [v. 4.0.5; 43]. Spatial calculations were performed with package sf [44], data distributions were visualized with *stat_slab* in the package ggdist [45], and data visualization was performed with package ggplot2 [46]. Locations were projected from the GPS native World Geodetic System 1984 (WGS84) to EPSG: 26,916, North American Datum 1983 (NAD83), Universal Transverse Mercator zone 16 N (NAD16) to allow for measuring distances and areas in meters. Other spatial layers were also transformed where necessary to match the same projection. The NOAA Medium Resolution 1:70,000 scale Digital Vector Shoreline [47] was used for maps. Bathymetry data were obtained from Digital Elevation Model (DEM) data from the NOAA Continuously Updated Digital Elevation Model (CUDEM) − 1/9 Arc-Second tiles [48]. Turtle locations were overlaid on the DEM and the Mean Sea Level (MSL) depth was assigned to each point. Hourly water level data from the National Oceanic and Atmospheric Administration (NOAA) station 8,729,108 in Panama City were then added to the elevation from the DEM to correct the water depth for each turtle telemetry location for current tide stage.

Telemetry locations with a horizontal dilution of precision > 25 were discarded. Speed and positional filters were then applied to further eliminate spurious locations such that any points where the travel speed was > 4 km per hour (km h^− 1^) were removed. Similarly, occasionally the tags would attempt an updated location if a poor-quality or unresolved QFP location was received. These duplicate locations, defined as points received within 10 min of each other, were identified, and the first location of each pair was discarded; the second location invariably being a higher-quality, updated location. The number of successful GPS locations per day was compared to the expected 24 attempts to measure daily success rate. Similarly, the number of successful locations each hour of the day compared to the number of days the transmitter was in operation allowed us to measure average success rate for daily time periods (e.g. day vs. night).

The point locations were converted to movement lines by joining successive points with line segments. The length of each line segment was calculated in meters by using the difference in UTM positions of the endpoints, the time to travel that distance was calculated as the difference between the GPS times of the endpoints, and travel speed was calculated by dividing distance by travel time. The polyline was then merged with the original point file so that the attributes of each travel line were assigned to the second of the two points used to draw the line. This second-point assignment associated the turtle’s destination with the travel paths.

Home range locations were determined by applying a kernel density function with standard deviation of 150 m to travel lines, filtered to a maximum speed of 0.08 km h^− 1^ and a maximum travel distance of 320 m, eliminating travel times > 4 h between successive locations. These were calculated with *density.psp* in the package spatstat [49]. Differences in travel rate were calculated using the function *lmer* and drifting rates were compared with a binomial model using function *glmer* (package lme4, [50]).

Preliminary observation of the turtle movements indicated behavioral shifts in several individuals that shifted near sunrise and sunset, so each 24 h period was split into day/night periods at the time of sunrise and sunset for each day as calculated by the function *sunrise* in the package maptools [51] for further analysis. We also explored changes in behavior during crepuscular times (here defined as two hours before to two hours after sunrise and sunset).

## Results

**Fig. 2:**
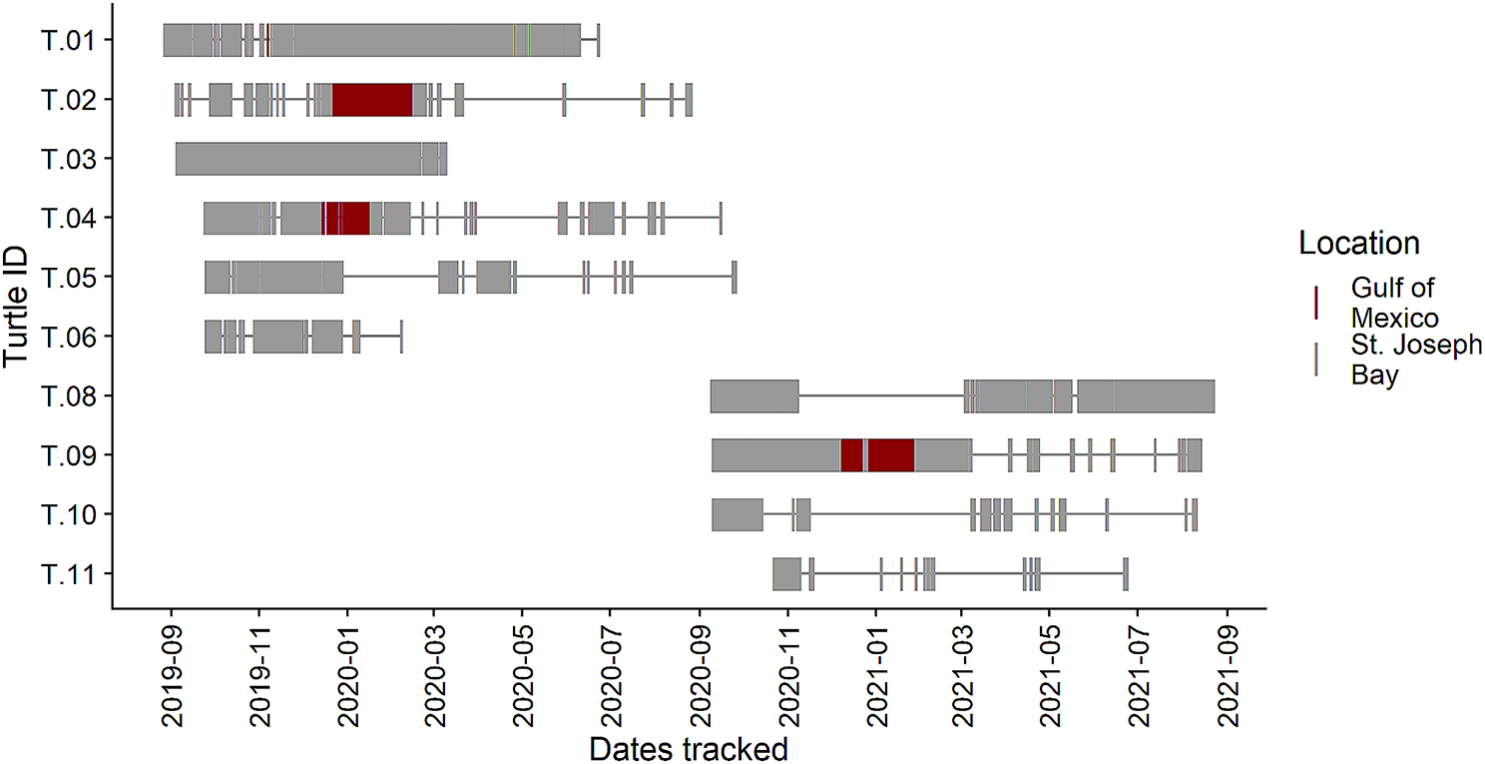
Timeline of locations received from 10 loggerhead sea turtles recorded hourly with GPS tags from September 2019 – September 2021

Of the eleven tracked loggerheads, nine were juveniles (< 80 cm CCL; [39, 52]) and two (T4 91.0 and T9 92.2 cm CCL) were most likely adults [53, 111]. All eleven turtles were tracked at various times during the study period which extended from September 2019 through September 2021 (Fig. 2). All turtles returned useable numbers of locations (536–4942 per individual), except.

one individual that was removed from the data set because only one month of data was received, with fewer than 100 locations. The final number of turtle GPS locations after filtering was 13,303, of which 12,203 were within St. Joseph Bay (Table 1). Most turtles stayed in the bay, but three individuals left the bay during winter and returned after 1–2 months in the Gulf. Another turtle briefly exited the mouth of the bay on multiple occasions but did not travel into open water (Fig. 1).

**Table 1:** Summary table

ID	CCL (cm)	Bay only	Habitat depths used	Total locations	Days tracked	Locations in SJB	50%ile home range (km^2^)	90%ile home range (km^2^)	Drifting episodes	Drifting % time
T.01	66.7	N	D	2,243	301	2,235	4.02	20.69	46	21.0
T.02	71.8	N	D	710	357	288	2.09	7.35	3	3.0
T.03	67.5	Y	DS	1,624	186	1,623	1.24	5.24	27	38.5
T.04	91.0	N	DS	1,134	317	845	2.98	11.11	6	5.6
T.05	70.8	Y	D	1,010	366	1,009	1.17	6.12	22	9.4
T.06	58.0	Y	DS	535	135	534	3.33	11.75	8	10.3
T.08	73.8	Y	DS	2,528	369	2,527	0.94	3.68	15	4.6
T.09	92.2	N	DS	2,615	337	2,240	2.67	11.90	26	9.7
T.10	70.3	Y	DS	641	334	640	0.52	2.64	11	4.5
T.11	75.2	Y	DS	263	244	262	1.10	4.11	2	1.4
*Mean*	*73.7*	*-*	*-*	*1,330.3*	*294.6*	*1,220.3*	*2.00*	*8.46*	*16.6*	*10.8*

D = Used deep habitat (> 5 m), S = used shallow habitat (< 1.5 m), SJB = St. Joseph Bay. CCL = curved carapace length. Home range values in km^2^, within St. Joseph Bay only

**Fig. 3:**
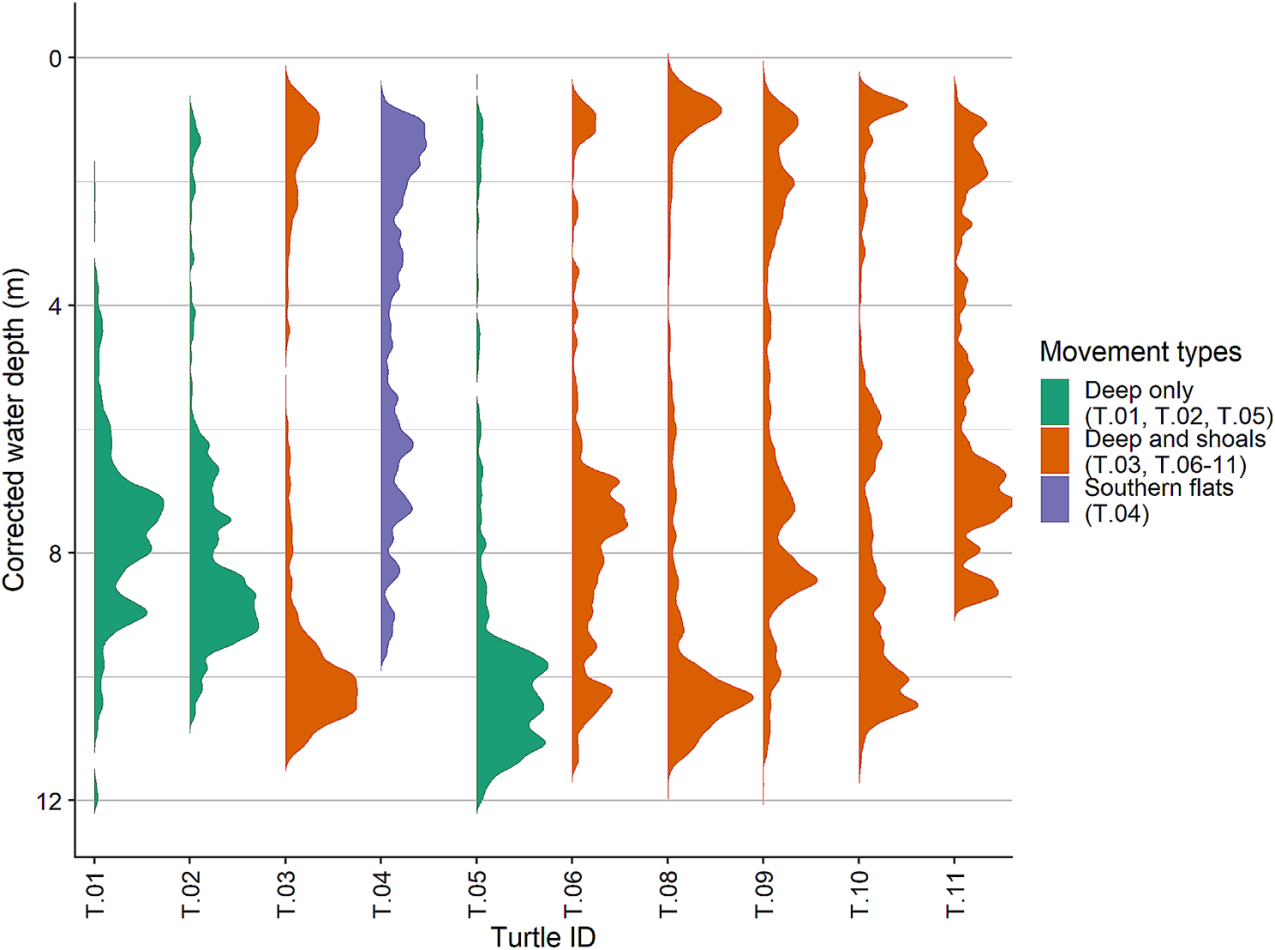
Individual density graph of water depths, corrected for tide stage of the locations from 10 loggerhead sea turtles recorded hourly with GPS tags from September 2019 – September 2021. Data from locations within St. Joseph Bay only

**Fig. 4:**
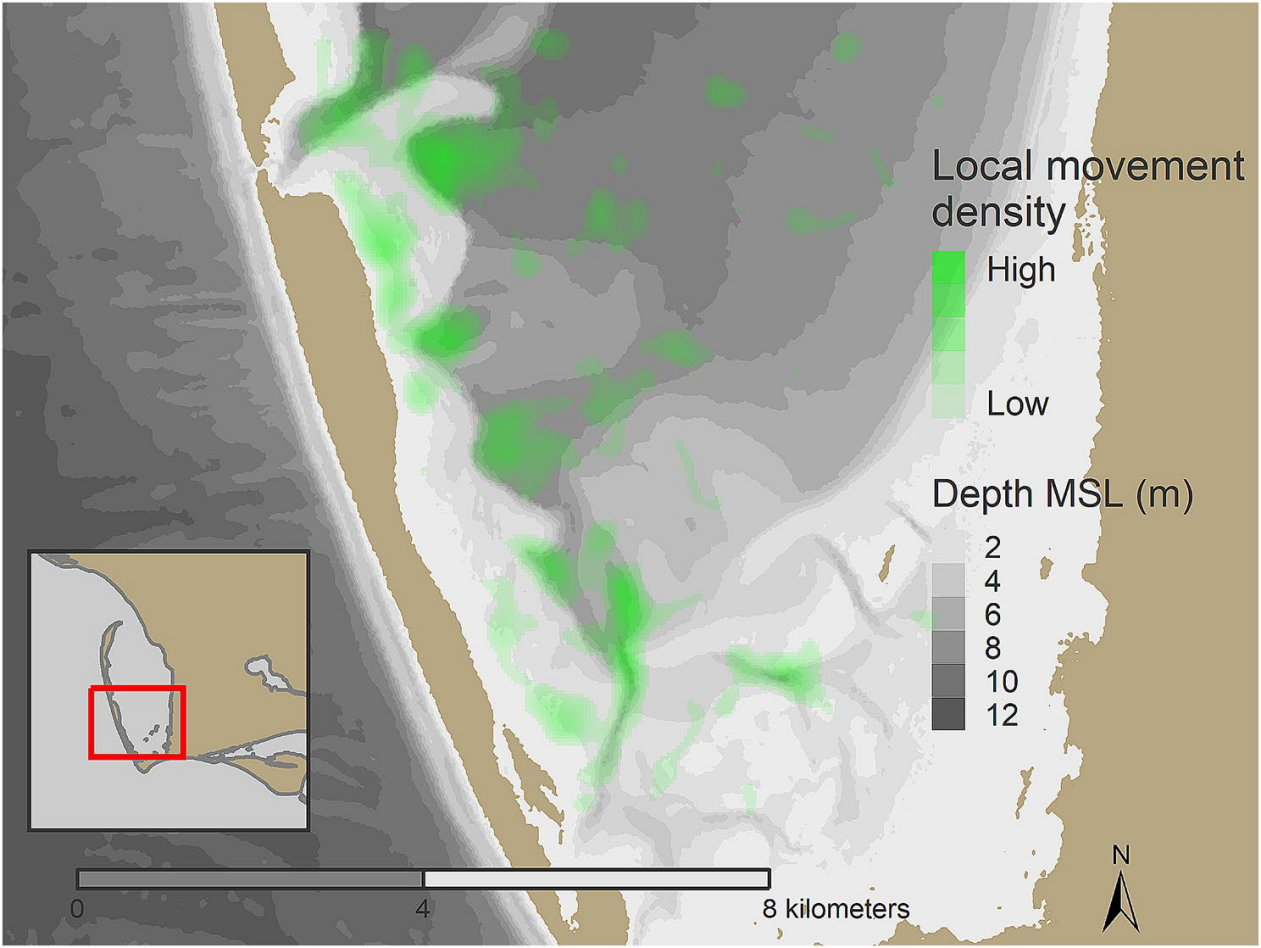
Kernel density plot of local, short-range movements from 10 loggerhead sea turtles recorded hourly with GPS tags from September 2019 – September 2021. Concentrations of habitat use in the west and southwest of St. Joseph Bay, Florida can be seen along shoal edges and within deeper channels, as well as along the tops pf shoals near the shoreline

Three of the tracked turtles remained in deep (> 5 m) water for almost all the tracking period, while all of the others made directed moves from deep water to shallow shoals (< 1.5 m) at regular intervals (Fig. 3). These turtles used shallow water habitats primarily at night. The deep-water locations used by turtles that made regular moves to shallow shoals were consistently at edges of shoals and in channels (Fig. 4).

**Fig. 5:**
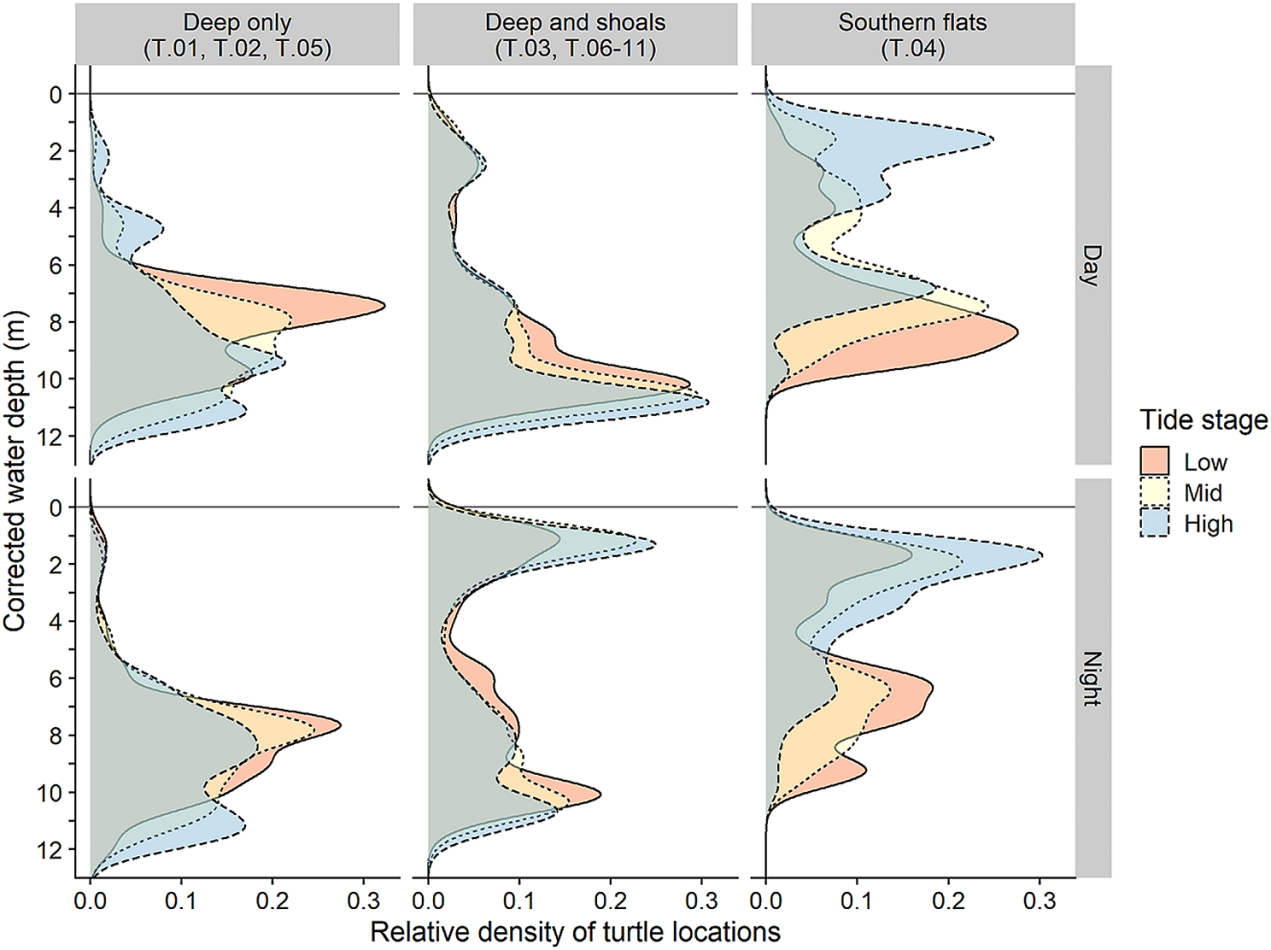
Combined density graph of water depths, corrected for tide stage of the locations from 10 loggerhead sea turtles recorded hourly with GPS tags from September 2019 – September 2021. Turtle movements were grouped by location and day/night, showing more use of shallow shoals at night, and at high tide stage

Movement rate for all of the tagged turtles was slightly faster during daylight hours compared to nighttime (Supplemental Fig.1). However, their home ranges were twice as large on average at night, indicating that they were selecting a wider variety of habitat patches during the hours of darkness. When crepuscular movements were considered, their movement speeds, habitat depths and locations were found to be intermediate, or mixtures of day and night measurements. Of the turtles that used shallow shoals, they moved closer to shore at night compared to the daytime, especially at high tide when access to the shoals was increased (Fig. 5; Supplemental Fig. 2). Shoal foraging depth averaged about 1 m at night, but the small amount of foraging on shoals that took place during the day was at about 2 m depth.

**Fig. 6:**
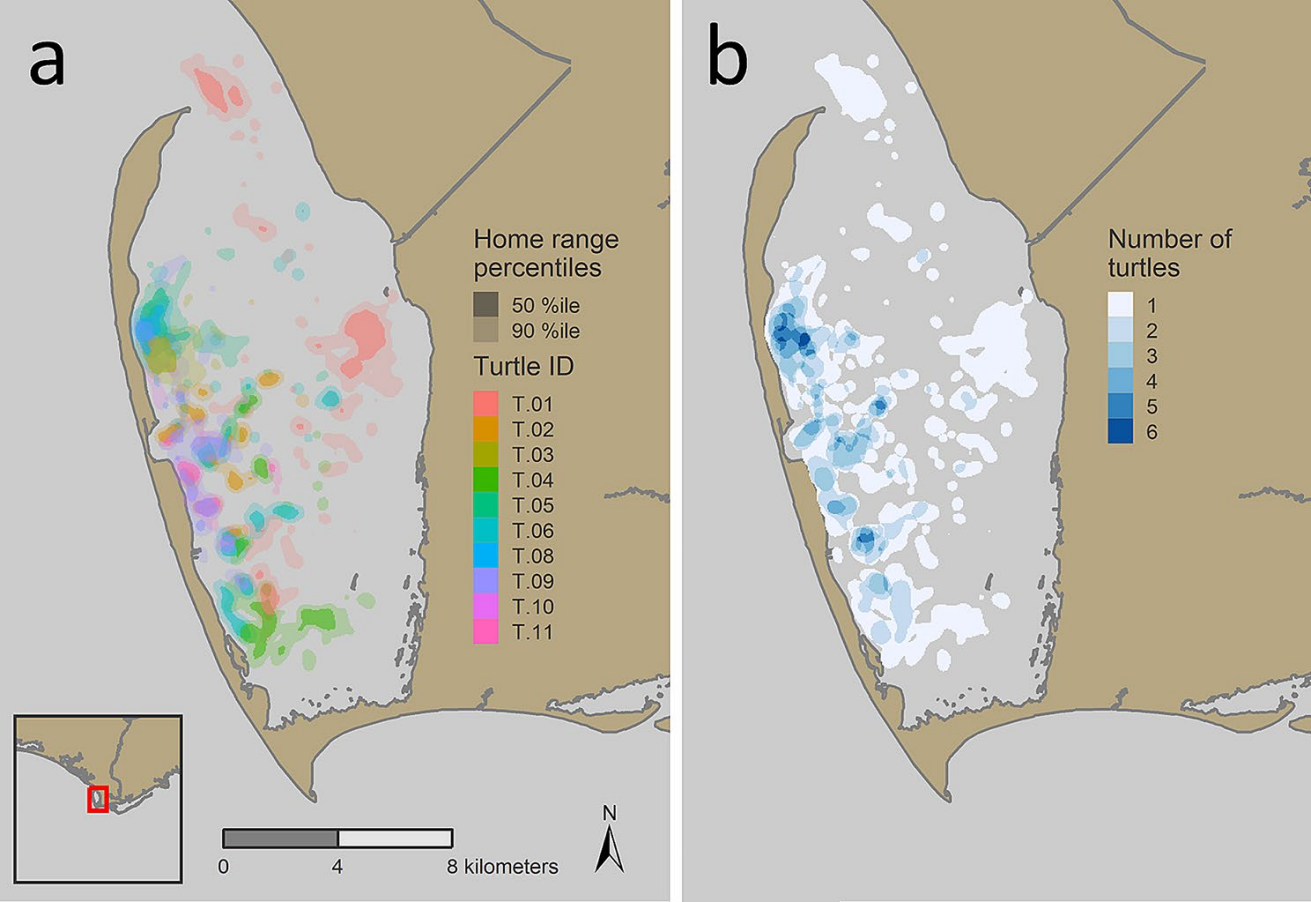
Kernel density plot of local, short-range movements from 10 loggerhead sea turtles recorded hourly with GPS tags from September 2019 – September 2021 within St. Joseph Bay, Florida. (**a**) Individual plots illustratingthe wide variety of individual home range locations, and (**b**) summed plots showing high use of the western shoreline

Home ranges for the loggerheads in St. Joseph Bay ranged from 0.52 to 4.02 km^2^ for the 50th percentile, and 2.64–20.69 km^2^ for the 90th percentile. The home range size was not related to the number of locations recorded, as the smallest home ranges were seen in turtles that recorded 262–2527 GPS locations, and the largest home ranges were seen in turtles that recorded 534–2235 GPS locations (Table 1). The home ranges showed very little overlap; instead, each turtle was located in distinct areas of the bay (Fig. 6) with 79% of the 50th percentile home ranges and 68% of the 90th percentile home ranges not overlapping with any other turtle (Table 2).

**Table 2:** Overlap of home ranges. Total area is the sum of all cells that are part of the home ranges of one or more turtles. Most home ranges did not overlap with those of other turtles

Percentile	# of Turtles	Total area (km^2^)	Percent of area
50%ile	1	12.56	78.81%
	2	2.64	16.57%
	3	0.74	4.62%
90%ile	1	36.77	67.58%
	2	9.54	17.53%
	3	4.92	9.03%
	4	2.12	3.90%
	5	0.87	1.59%
	6	0.20	0.37%

GPS location relay success rate was ~ 20–80% less during the day compared to night, especially in the morning hours from 8 to 12. This timing is similar to deep water to shallow shoals moves among those turtles that made those moves. The amount of data recovered after the initial transmission attempt with the Iridium re-transmission ability ranged from as little as ~ 5% to over 80%, recovering data from up to three months in the past. There was no apparent pattern in re-transmission related to turtle size or movement pattern: we suspect it was related to how high each transmitter antenna rode above the water surface, which depended on each turtle’s swimming attitude.

The median travel speed of the turtles within St. Joseph Bay was 0.046 km h^− 1^ (95% range 0.004–0.714). In the open Gulf, their speed was considerably faster, with a median of 0.238 km h^− 1^ (95% range 0.004–1.530; t = 17.1, *p* < 0.001; Supplemental Fig. 1). Within the bay, turtles traveled faster in water depths < 6 m (median 0.105 km h^− 1^; 95% range 0.006–0.740) than they did in deeper water (median 0.036 km h^− 1^; 95% range 0.004–0.684; t = 30.4, *p* < 0.001; Supplemental Fig. 1). They also travelled faster during the day (median 0.072 km h^− 1^; 95% range 0.005–1.026) vs. at night (median 0.038 km h^− 1^; 95% range 0.004–0.794; t = 21.9, *p* < 0.001). Warmer water temperatures were associated with higher travel speeds as well, with a 0.037 km h^− 1^ (SE = 0.003) increase in speed for each degree C (t = 13.6, *p* < 0.001; Supplemental Fig. 1).

**Fig. 7:**
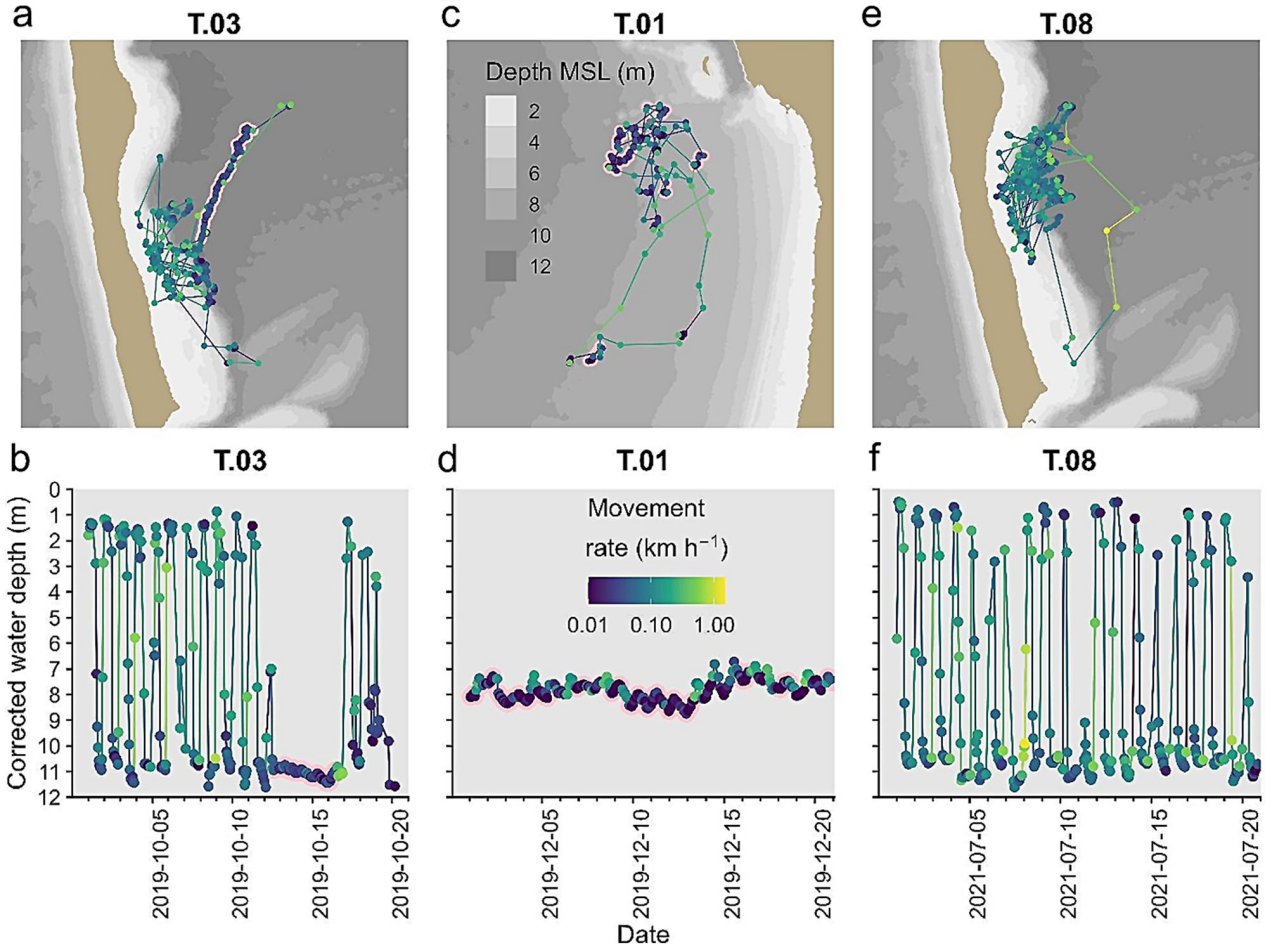
Travel maps and time series plots of loggerhead sea turtles recorded hourly with GPS tags from September 2019 – September 2021 highlighting a novel behavior we termed “drifting”. Panels a, c, and e show spatial movements of three turtles, and panels b, d and f show the corrected water depth of each turtle over time. The color of the points and lines indicate the speed of the turtle, and pink outlines highlight when the turtle was drifting. (**a**,**b**) Turtle T.03 showing daily movements to shallow shoals during high tide at night, alternating with periods of offshore deep-water drifting; (**c**,**d**) turtle T.01 spent its time in deep water, alternating between periods of activity and drifting, (**e**,**f**) turtle T.08 used shallow shoals mostly at night at high tide, similar to turtle T.03 but rarely showed drifting behavior

While examining the movement data we encountered a recurring long-term slow movement we are calling “drifting” (Fig. 7). It was characterized by at least 12 h (up to 5 days in duration) of continuous slow movements (0.06 kmh-1) that upon visual review had small turning angles (< 20°), and high GPS reception rates (at least 50% successful). All of the turtles exhibited drifting behavior, but there was a wide range of prevalence among the turtles, from 1.4% of their time in only two identified drifting episodes (T-11) to 38.5% of their time in 46 episodes (T-03; Table 1). There was no apparent correspondence between habitat use behaviors (deep only vs. deep and shallow) and drifting behavior, but colder water temperatures were associated with more drifting (e.g. 36.2% below 16 °C vs. 4.7% above 28 °C; *glmer* z = 42.5, *p* < 0.001).

## Discussion

Globally, loggerheads exhibit a dichotomy in basin-wide movement patterns with some individuals foraging in oceanic waters and others in neritic habitats [54–57]. Similar variation was also evident on the fine scale at a foraging area in St. Joseph Bay where juvenile and adult loggerheads exhibited three general movement patterns: (1) nocturnal movements onto shallow seagrass shoals during high tide, (2) consistent use of deeper waters, sometimes immediately adjacent to those shoals, and (3) use of shallow seagrass flats in the southern end of the bay during high tide. Some of these movement patterns were similar to those reported by Dujon et al. [1] from the Mediterranean Sea, and in both their study and ours, movement patterns varied by time of day, water depth and temperature. However, the movements we documented occurred on a relatively small spatial scale (i.e., within one coastal bay vs. within a sea or ocean basin). Additionally, our tagged loggerheads displayed some novel movements such as periodically drifting in deeper waters for multiple days, which they did more of in colder water temperatures (Fig. 7). Tagged loggerheads in our study established relatively small home ranges [1, 4, 58], similar in size to, but slightly smaller than, those reported by Lamont and Iverson [13], that overlapped only minimally among individuals. As has been documented with other sea turtle studies [19, 21, 59], the GPS tags used here provided us with relatively long-term and fine-scale movement data for adult and juvenile loggerheads.

It is likely that fixed foraging strategies have developed among individual loggerheads, as has been reported elsewhere [1, 56], that have resulted in different movement patterns. Individual variability in movement patterns exist across taxa and habitats [60, 61] and can reflect long-term behaviors (i.e., individual personality or life-history strategies; [56]) or short-term behavioral plasticity in response to changing environmental variables [60, 62, 63]. This variability can contribute to niche specialization [64]. As a species, loggerheads are considered generalist carnivores with a relatively large foraging niche [65] however individual specialization in diet has been documented [54, 66]. In St. Joseph Bay, individuals that used shallow shoals and deeper waters were most likely targeting benthic invertebrates (e.g., gastropods, crustaceans) whereas those that remained in deep water were either undertaking dives to the benthos to forage [62, 67, 68] or foraging on floating invertebrates such as tunicates and jellyfish [68, 69]. Individuals that remained in deep water were often located immediately adjacent to shoals but never actually moved onto those shallow-water habitats which suggests this is an individual strategy [56, 70] or there is a force such as intraspecific competition [1, 71] keeping those individuals off the shoals. In the eastern Gulf of Mexico, Silver-Gorges et al. [72] suggested adult loggerheads foraged and resided in shallow habitats, and juveniles moved between deeper locations to forage and shallower locations to rest. In their study, some juveniles however, also foraged and resided in shallow areas, adjusting behaviors to avoid intraspecific competition [72]. Similar movement patterns among adult and juvenile loggerheads were not documented at our site, however intraspecific competition could still occur [71]. In fact, home ranges of our tagged loggerheads only minimally overlapped which may suggest intraspecific competition (Table 1). Additionally, green turtle and Kemp’s ridley home ranges overlap with loggerheads in St. Joseph Bay [13] and interspecific competition may also impact loggerhead movement patterns [71].

For loggerheads that undertook movements between shallow shoals and deeper waters, predation risk most likely drove the diel pattern where turtles used deep waters during the day and shallow waters at night. Similar movement patterns have been observed in fish [73] and elasmobranchs [74]. Tiger sharks are known predators of sea turtles [6], are more active in the Gulf of Mexico during the day [75] and are present in St. Joseph Bay. In fact, we have observed tiger sharks foraging on sea turtles in shallow seagrass habitat in the bay. Although use of different diurnal home ranges has been reported in various sea turtle species, typically individuals used deeper refuges at night and shallower foraging habitats during the day [70, 76] which is in opposition to what we documented for our tagged loggerheads. Loggerheads in the Mediterranean exhibited a dichotomy in diurnal habitat use, with some individuals using deep waters at night and others using shallow night-time refuges [1]. Dujon et al. [1] suggested these differences reflected the availability of refuges (e.g., bathymetry, availability of structures, etc.) to avoid predators at each site. The lack of refuges (i.e., reefs and ledges; but see [71]) in St. Joseph Bay may drive the diurnal movement patterns documented here, as opposed to the pattern suggested by Silver-Gorges et al. [72] in the eastern Gulf of Mexico (e.g., adults resided in shallow waters) where reefs are more prevalent [77]. Our tracked loggerheads, regardless of foraging strategy, moved more at night than during the day. Their home ranges were larger at night, indicating greater activity and further suggesting turtles were taking refuge during the day and foraging at night. Tags used in our study did not provide dive data for the turtles as has been reported in other studies using depth logging tags (e.g., Time Depth Recorders; [78–80] or satellite tags that contain depth sensors [81–83]. Direct measures of dive behavior from loggerheads would further elucidate these movement patterns, particularly for deep loggerheads [82, 84, 85].

In addition to general movement patterns and diurnal relationships, the loggerheads we tracked exhibited a behavior we termed drifting. These movements occurred both night and day, almost exclusively over deep water and lasted for multiple days. Although not frequently reported, studies have documented a similar surface behavior commonly termed basking however basking is typically short-term (e.g., hours and not days; [79, 86]). Basking has often been suggested as a thermoregulatory action in response to cold temperatures [87, 88] or deep dives below the thermocline [89, 90] which may enhance the digestive process [91, 92]. In our study drifting was more common in colder water temperatures but occurred in all temperatures and seasons (see also [79]), however if there is an optimal temperature at which loggerhead digestive rates are maximized, individuals may exhibit behaviors throughout the year to maintain that optimal temperature regardless of sea surface temperature [92, 93]. For example, in summer when water temperatures rise above 32 °C loggerheads may drift in deeper waters to (1) reduce energy expenditure and (2) move out of warmer, shallow waters [92, 94]. Alternatively in winter, turtles may bask at the surface to increase body temperatures [88].

Home ranges of our tracked loggerheads were small relative to those reported for loggerheads at other sites throughout the world [37, 57, 58, 68, 95], for nesting loggerheads from the Gulf of Mexico [4, 96] and for those previously reported in St. Joseph and St. Andrews Bays, FL [13]. However, location accuracy can greatly impact estimates of home range size [12] and as such, use of GPS technology with small positional error rates relative to Argos-only tags allows the use of a relatively small convolution kernel and likely results in smaller home range estimates [15, 95, 97]. For example, using data from GPS tags [1], documented relatively small home ranges for loggerheads in offshore (90.2 km^2^) and nearshore (24.3 km^2^) waters in the Mediterranean. Alternatively, home ranges for loggerheads tracked across the Mediterranean using Argos satellite tags were much larger [32, 95, 98]. We documented no difference in home range sizes relative to movement patterns (i.e., deep vs. shallow-deep). Although the largest home range we documented was a turtle that used deep-water (T.01), the two remaining deep turtles (T.02, T.05) used relatively small home ranges (Table 1). This is in opposition to Dujon et al. [1]. who found offshore home ranges were larger than nearshore home ranges and suggested turtles had to search broader areas in deeper waters for prey. While there were no differences in offshore/nearshore home ranges in St. Joseph Bay, daytime home ranges were much smaller than nighttime home ranges suggesting turtles were more active at night. This is in opposition to diurnal home range sizes reported for green turtles (Christiansen et al. 2017) and hawksbills (Hart et al. 2012, Wood et al. 2017) that suggested turtles rested at night and were active during the day (Hays et al. 2024). Our nocturnal home range sizes support our data that showed some turtles moved onto shoals at night, most likely to forage. Additionally, our tags transmitted a lower rate of satellite communications during the day, further suggesting that turtles exhibited deep water resting behavior during the day resulting in relatively small home ranges and active foraging at night over larger home ranges.

Three of the 10 tracked loggerheads left St. Joseph Bay for approximately 1–2 months when water temperatures dropped in winter. Those three turtles primarily remained in the Gulf of Mexico immediately adjacent to St. Joseph Bay; one undertook a foray of approximately 120 km to the southeast of the bay. Sea turtles inhabiting temperate regions frequently move out of coastal bays in winter to avoid cold temperatures [13, 99, 100]. Individuals that remain in coastal bays risk cold-stunning [101, 102]. Our tagged turtles traveled fastest while in the Gulf of Mexico, which is not surprising as oceanic movements are usually faster than coastal movements [103–105]. However, compared to travel speeds reported for loggerheads during migration (e.g., 0.14–0.36 m s^− 1^ or 0.50–1.30 km h^− 1^ in [105]), travel speeds for loggerheads outside of St. Joseph Bay (0.0036–1.53 km h^− 1^) were comparable, while speeds within the bay (0.0036–0.713 km h^− 1^) were slow [105–107]. While in the bay, fastest travel speeds occurred at intermediate water depths, which suggests turtles were traveling between benthic foraging or resting locations and shallow foraging areas [104, 105].

## Conclusions

Compared to the more commonly used Argos satellite tags [33, 108], the GPS tags use here were ideal for use on an organism such as a sea turtle that spends little time at the ocean’s surface. The ability of tags to backfill data when turtles are at the surface for long periods of time provides valuable location information that would otherwise be lost to sea turtle tracking studies. In our study, data from two turtles would have been removed due to insufficient number of transmissions, and overall precision from all tags would have declined without the back-filled information.

Our data showed a clear division in habitat use between loggerheads that used only deep waters and those that moved from shallow shoals to deeper waters and highlights the need to look more closely at diving and foraging patterns of these individuals. If these individuals are foraging on separate prey items, there may also be morphological differences, such as variations in head width [109] or body condition [110] between loggerheads that exhibited different movement patterns. Finally, only three of our tracked turtles remained in deep waters throughout the tracking period. This is notable because our capture methods, which require visual location and hand capture of turtles, are only effective in water depths less than approximately 4 m, into which deep turtles rarely move. Continued use of these GPS tags with loggerheads inhabiting coastal foraging areas could confirm whether the movement patterns we documented in St. Joseph Bay occur elsewhere or only at our study site and could help elucidate drivers of these different patterns.

### Electronic Supplementary Material

Below is the link to the electronic supplementary material.

Supplementary Material 1

## Data Availability

Data have been deposited in ScienceBase and can be accessed at https://doi.org/10.5066/P13TCY7X.

## Declarations

### Disclaimer

Any use of trade, firm, or product names is for descriptive purposes only and does not imply endorsement by the US Government.
