# Supplementary material for: Deep vs shallow: GPS tags reveal a dichotomy in movement patterns of loggerhead turtles foraging in a coastal bay
Source: Mov Ecol. 2024 May 30;12:40. doi: 10.1186/s40462-024-00480-y (PMC11140867; doi:10.1186/s40462-024-00480-y)
Supplement: Supplementary file 1 — Supplementary Material 1 [file 40462_2024_480_MOESM1_ESM.docx]

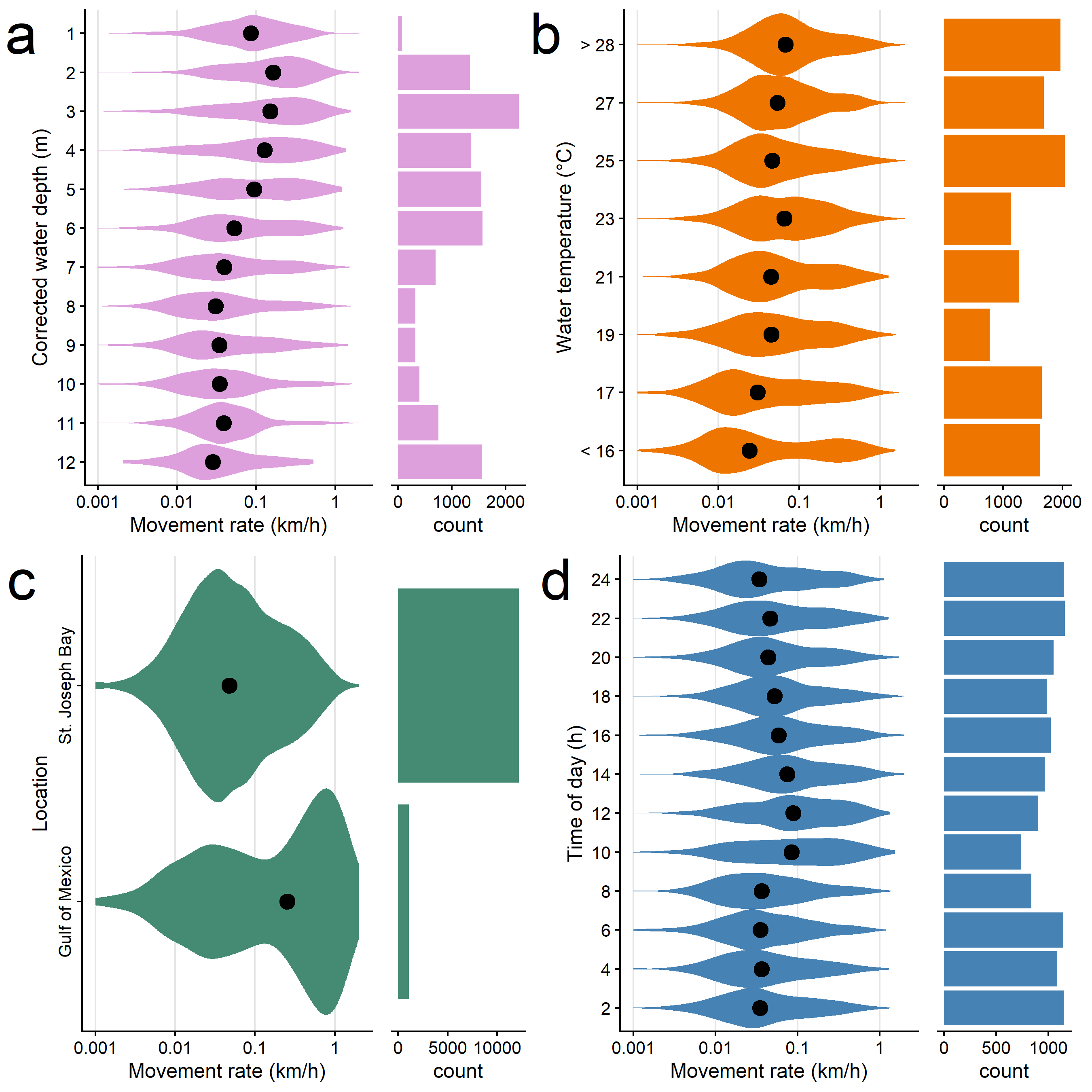


Supplemental Figure 1: Violin plots of travel speed calculations along with sample sizes (on right in each panel) from 10 loggerhead sea turtles recorded hourly with Iridium-linked GPS tags from September 2019 – September 2021 within St. Joseph Bay, Florida. Black points represent the median for each grouping. a) travel speed based on water column depth at each location, corrected for tide, b) travel speed based on water temperature, c) travel speed based on location (within St. Joseph Bay or outside the bay), and d) travel speed based on hour of the day.


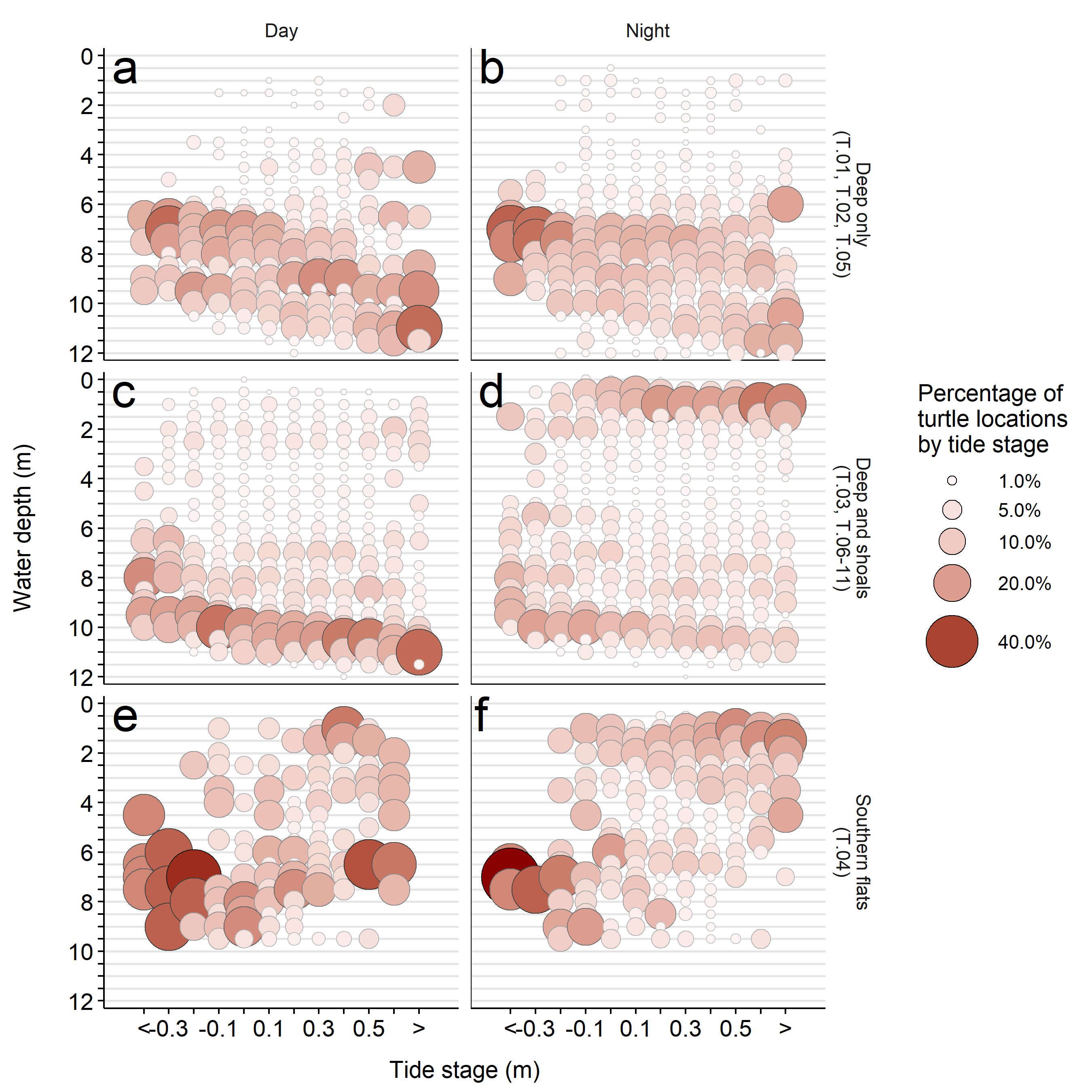


Supplemental Figure 2:Bubble plot of water depth vs tide stage for lcations from 10 loggerhead sea turtles recorded hourly with Iridium-linked GPS tags from September 2019 – September 2021 within St. Joseph Bay, Florida. Larger and Darker circles indicate more prevalent water depth for each tide stage value.
